# Oral Leucine Supplementation Is Sensed by the Brain but neither Reduces Food Intake nor Induces an Anorectic Pattern of Gene Expression in the Hypothalamus

**DOI:** 10.1371/journal.pone.0084094

**Published:** 2013-12-13

**Authors:** Thais T. Zampieri, João A. B. Pedroso, Isadora C. Furigo, Julio Tirapegui, Jose Donato

**Affiliations:** 1 Department of Physiology and Biophysics, Institute of Biomedical Sciences, University of São Paulo, São Paulo, Brazil; 2 Department of Food Science and Experimental Nutrition, Faculty of Pharmaceutical Sciences, University of São Paulo, São Paulo, Brazil; University of Santiago de Compostela School of Medicine - CIMUS, Spain

## Abstract

Leucine activates the intracellular mammalian target of the rapamycin (mTOR) pathway, and hypothalamic mTOR signaling regulates food intake. Although central infusion of leucine reduces food intake, it is still uncertain whether oral leucine supplementation is able to affect the hypothalamic circuits that control energy balance. We observed increased phosphorylation of p70s6k in the mouse hypothalamus after an acute oral gavage of leucine. We then assessed whether acute oral gavage of leucine induces the activation of neurons in several hypothalamic nuclei and in the brainstem. Leucine did not induce the expression of Fos in hypothalamic nuclei, but it increased the number of Fos-immunoreactive neurons in the area postrema. In addition, oral gavage of leucine acutely increased the 24 h food intake of mice. Nonetheless, chronic leucine supplementation in the drinking water did not change the food intake and the weight gain of *ob/ob* mice and of wild-type mice consuming a low- or a high-fat diet. We assessed the hypothalamic gene expression and observed that leucine supplementation increased the expression of enzymes (BCAT1, BCAT2 and BCKDK) that metabolize branched-chain amino acids. Despite these effects, leucine supplementation did not induce an anorectic pattern of gene expression in the hypothalamus. In conclusion, our data show that the brain is able to sense oral leucine intake. However, the food intake is not modified by chronic oral leucine supplementation. These results question the possible efficacy of leucine supplementation as an appetite suppressant to treat obesity.

## Introduction

 The regulation of the energy balance and food intake relies on the ability of the central nervous system (CNS) to receive and process information about the nutritional status of the organism. This information is conveyed by hormones, such as leptin, insulin and ghrelin. In addition, variations in the circulating levels of nutrients also convey important information to the CNS about the fed/fasting state [1,2]. It has long been known that specific populations of neurons can sense glucose levels. Glucose-sensing neurons play a key role in the control of glucose homeostasis, energy balance and counterregulatory responses to hypoglycemia [3-5]. There have also been reports indicating that the brain has a lipid-sensing ability that is critical for the control of energy balance and insulin sensitivity [6,7]. However, despite the fact that amino acids are indispensable macronutrients as substrates for the synthesis of proteins and other molecules, much less is known about the ability of the brain to sense their circulating levels. In fact, the body seems to detect variations in the concentrations of amino acids because systemic administration of amino acids can stimulate whole-body protein synthesis [8,9]. Among all amino acids, it appears that the branched-chain amino acid (BCAA) leucine is of particular importance in conveying the level of amino acid availability to cells because leucine is the most potent amino acid that activates the mammalian target of the rapamycin (mTOR) intracellular signaling pathway, which is critical for initiating the protein translation process [10-16].

The availability of leucine has effects beyond the control of protein synthesis, and some studies have also demonstrated that supplementation with leucine induces changes in energy balance and adiposity. Thus, it has been speculated that leucine supplementation could be used for the treatment and prevention of obesity [17,18]. However, previously obtained results have been controversial. Leucine supplementation decreases adiposity in food-restricted rats [19] and during aging [20]. However, while some studies have shown that leucine supplementation reduces diet-induced obesity in rodents [21-25], others did not find any significant effects on adiposity with leucine supplementation [26,27]. In humans, the combined supplementation of leucine and pyridoxine (vitamin B6) increased the fat oxidation of overweight subjects [28]. However, 3 months of leucine supplementation did not change the weight, the body composition and the energy intake and macronutrient composition, calculated from the dietary intake records, of healthy elderly men [29].

It is unclear how leucine is able to influence the energy balance. Cota et al. [30] showed that hypothalamic mTOR signaling regulates food intake. As leucine is a natural activator of the mTOR signaling pathway, it is plausible to hypothesize that leucine supplementation recruits mTOR signaling in the hypothalamus, which in turn causes a reduction in food intake. However, the vast majority of the studies that assessed the consequences of leucine supplementation did not find any reduction in food intake, even those studies that observed decreases in body fat mass [19-21,24-27,31-37]. On the other hand, when leucine is directly administered in the CNS through an intracerebroventricular (i.c.v.) cannula, it does indeed cause a reduction in food intake [22,30,38,39]. Considering that oral, instead of central, administration of leucine is the only feasible and physiological way to supplement leucine in people, it is imperative to clarify whether oral leucine supplementation is in fact able to affect hypothalamic circuitries that regulate food intake. Therefore, the objective of the present study was to employ acute and chronic paradigms of oral leucine supplementation to assess possible changes in food intake and activation of hypothalamic components that control energy balance.

## Results

### Experiment 1

#### Acute oral administration of leucine induces phosphorylation of p70S6K in the hypothalamus

**Figure 1:**
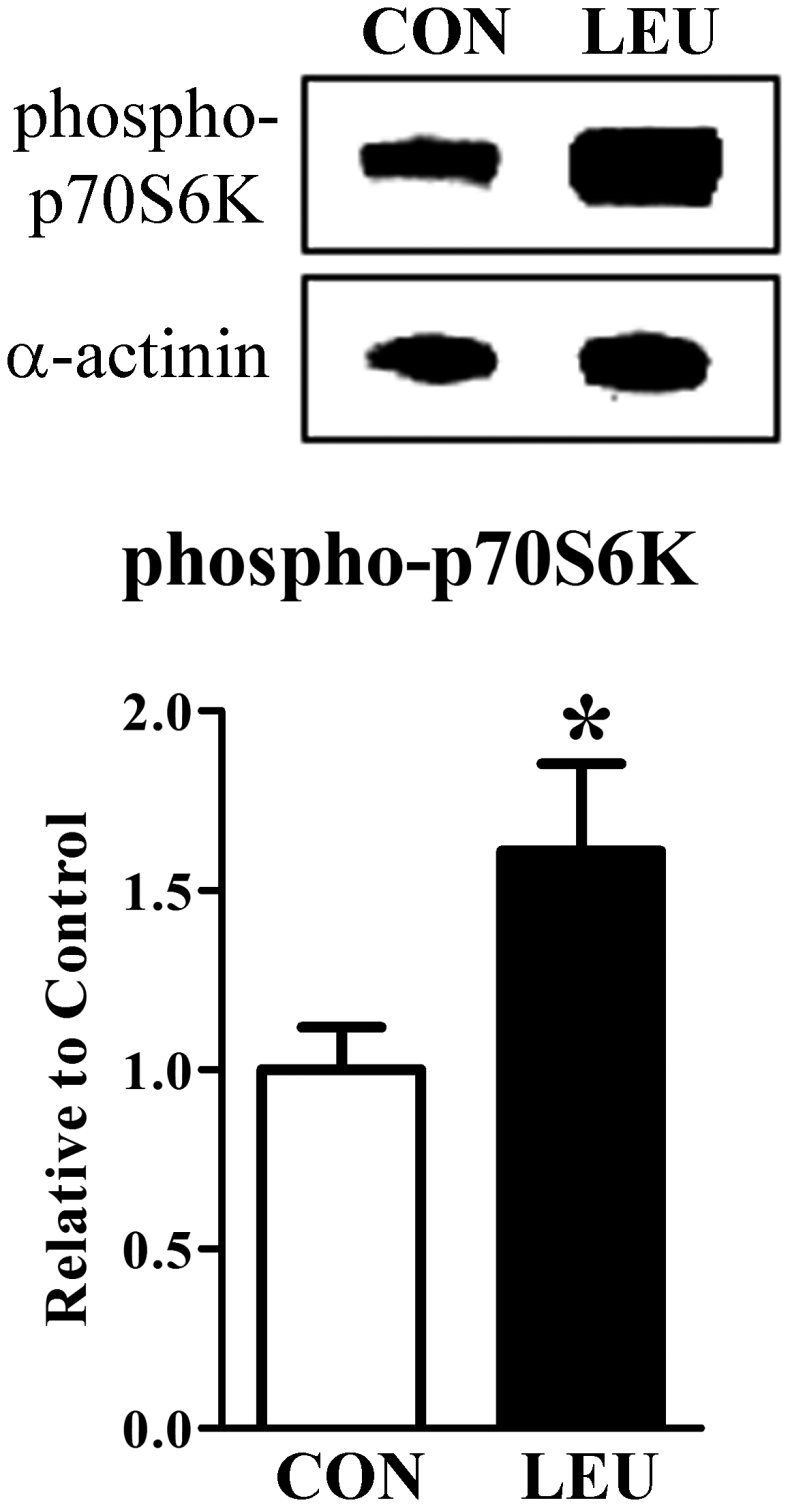
Acute oral leucine administration induced phosphorylation of p70S6K in the hypothalamus. Bar graphs showing the quantification of the phosphorylation of p70S6K in the hypothalamus of mice that received oral gavage of water (CON group, *n* = 9) or leucine solution (LEU group, *n* = 9). The data were normalized to the expression of α-actinin. *, significantly different (*P* < 0.05) from the CON group.

 Acute oral administration of leucine causes activation of p70S6K in the skeletal muscle [10,11], the liver [12,40] and white adipose tissue [14,41]. In the hypothalamus, p70S6K phosphorylation can be induced by i.c.v. administration of leucine [22,30]. However, it is still uncertain whether orally administered leucine is able to recruit this signaling pathway in the hypothalamus. We observed that orally administered leucine increased the phosphorylation of p70S6K in the hypothalamus of mice (Figure 1).

### Experiment 2

#### Acute oral administration of leucine induces the expression of Fos protein in the area postrema

**Figure 2:**
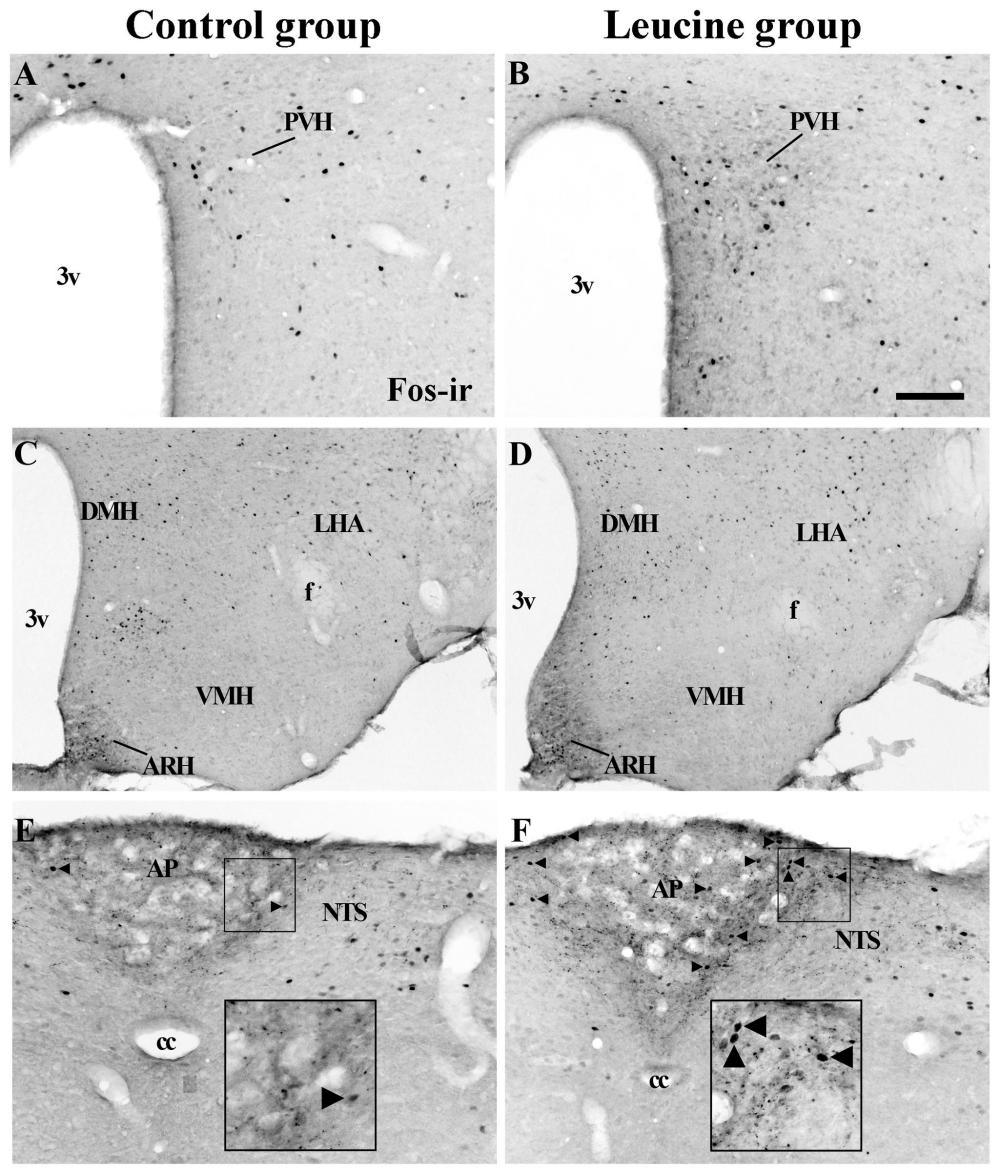
Acute oral leucine increased the expression of Fos in the area postrema. A-F. Brightfield photomicrographs of mouse brain sections showing the Fos immunoreactivity (Fos-ir) in the paraventricular nucleus of the hypothalamus (PVH; A-B), the mediobasal hypothalamus (C-D), the nucleus of the solitary tract (NTS; E-F) and the area postrema (AP; E-F) of mice from control group (A, C, E; *n* = 10) and leucine group (B, D, F; *n* = 9). Arrowheads indicate neurons expressing Fos-ir in the AP. The boxes shown in E and F exhibit a higher magnification image in the AP. Abbreviations: 3v, third ventricle; ARH, arcuate nucleus of the hypothalamus; cc, central channel; DMH, dorsomedial nucleus of the hypothalamus; f, fornix; LHA, lateral hypothalamic area; VMH, ventromedial nucleus of the hypothalamus. Scale Bar: A-B = 50 µm; C-D = 100 µm; E-F = 50 µm (higher magnification boxes = 25 µm).

 The expression of Fos protein is a well-established acute marker of activated neurons [42]. Therefore, we assessed whether oral leucine administration is able to activate neurons in several nuclei of the hypothalamus and brainstem related to the regulation of the energy balance (Table 1). In the hypothalamus, the number of neurons expressing Fos-immunoreactivity (Fos-ir) in the paraventricular nucleus of the hypothalamus (PVH, Figure 2A-B), the arcuate nucleus of the hypothalamus (ARH, Figure 2C-D), the ventromedial nucleus of the hypothalamus (VMH, Figure 2C-D), the lateral hypothalamic area (LHA, Figure 2C-D) and the dorsomedial nucleus of the hypothalamus (DMH, Figure 2C-D) were similar between the leucine and control groups (Table 1). In the brainstem, we observed that the area postrema (AP) of leucine-treated mice showed a higher number of Fos-expressing neurons than the control group (Figure 2E-F and Table 1). No difference in the expression of Fos-ir in the nucleus of the solitary tract (NTS) was observed between groups (Figure 2E-F and Table 1).

**Table 1:** Number of neurons expressing Fos immunoreactivity in nuclei of the hypothalamus and brainstem of mice from control and leucine groups (*n* = 9-10 per group).

**Area**	**Control Group**	**Leucine Group**	***P***
PVH	6.1 ± 1.6	8.0 ± 1.4	0.3913
ARH	9.4 ± 2.2	9.9 ± 2.4	0.8806
VMH	10.4 ± 2.2	12.8 ± 2.3	0.4669
LHA	24.9 ± 3.3	23.7 ± 2.7	0.7811
DMH	22.8 ± 1.4	18.8 ± 3.9	0.3246
NTS	3.7 ± 0.8	3.4 ± 0.8	0.8216
AP	1.5 ± 0.4	11.6 ± 2.6*	0.0008

^*^ significantly different (*P* < 0.05) from control group (Student’s t-test). AP, area postrema; ARH, arcuate nucleus of the hypothalamus; DMH, dorsomedial nucleus of the hypothalamus; LHA, lateral hypothalamic area; NTS, nucleus of the solitary tract; PVH, paraventricular nucleus of the hypothalamus; VMH, ventromedial nucleus of the hypothalamus.

### Experiment 3

#### Oral administration of leucine acutely increased the food intake of mice

**Figure 3:**
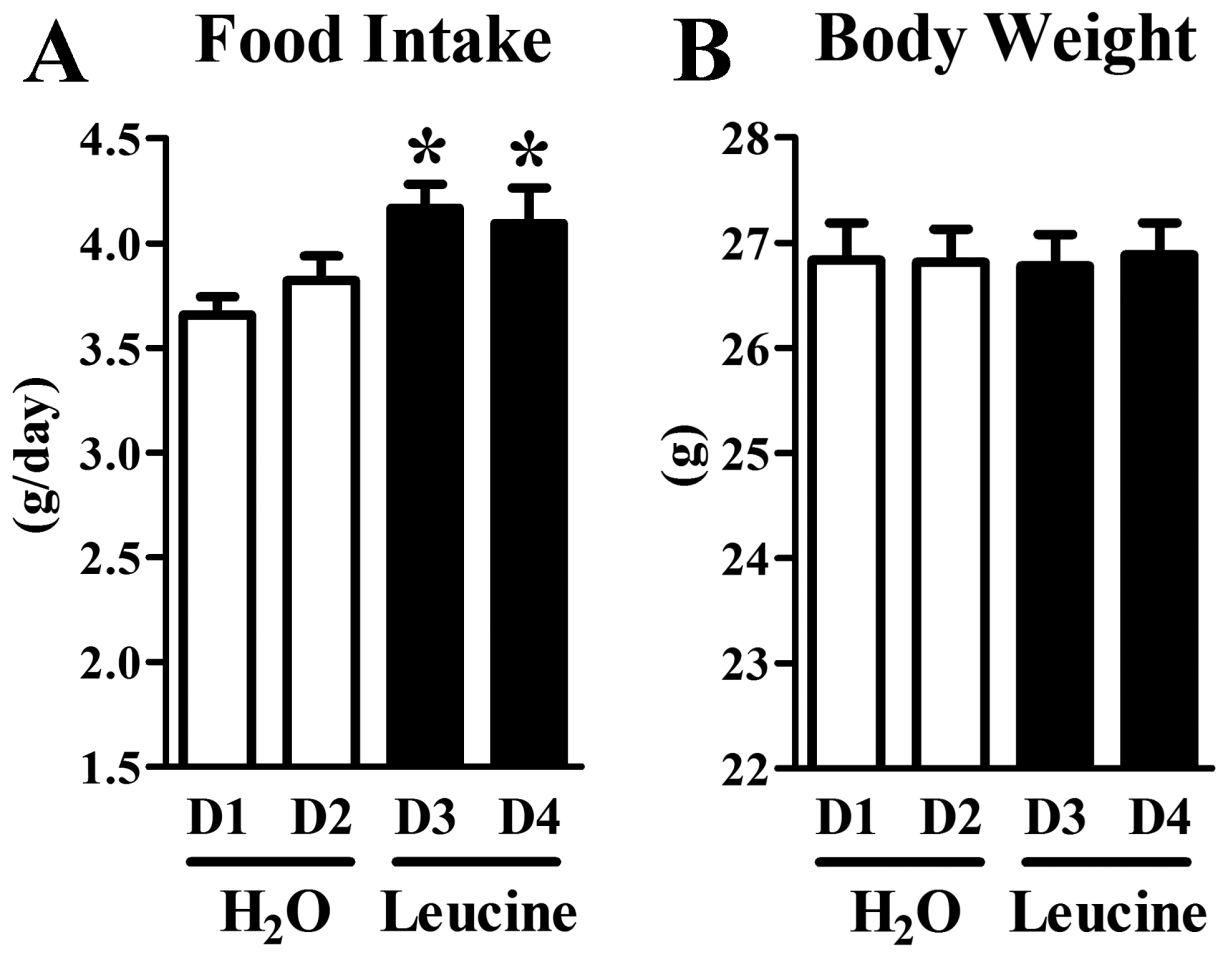
Oral Leucine supplementation acutely increased food intake. A. Oral gavage of a leucine solution increased the 24 h food intake (Day 3 and Day 4) compared to the days when the animals received gavage of water (Day 1 and Day 2). B. No changes in body weight were observed among the experimental days. *, significantly different (*P* < 0.05) from D1 and D2 (gavage of water).

The previous results suggested that the brain is responsive to oral leucine supplementation. To determine whether acute oral administration of leucine produces changes in the food intake we daily habituated 20 single-housed mice to receive 0.5 mL of water through gavage for one week. Latter, mice received a gavage containing leucine for two days. Their food intake and weight gain were compared to that observed after receiving only water. Surprisingly, we observed an increased food intake in the days when the mice received leucine in comparison with the control (water) days (Figure 3A). No changes in the weight gain (data not shown) or body weight (Figure 3B) were observed among the studied days. 

### Experiment 4

#### Chronic leucine supplementation in the drinking water does not change the food intake of genetically obese (*ob/ob*) mice

**Figure 4:**
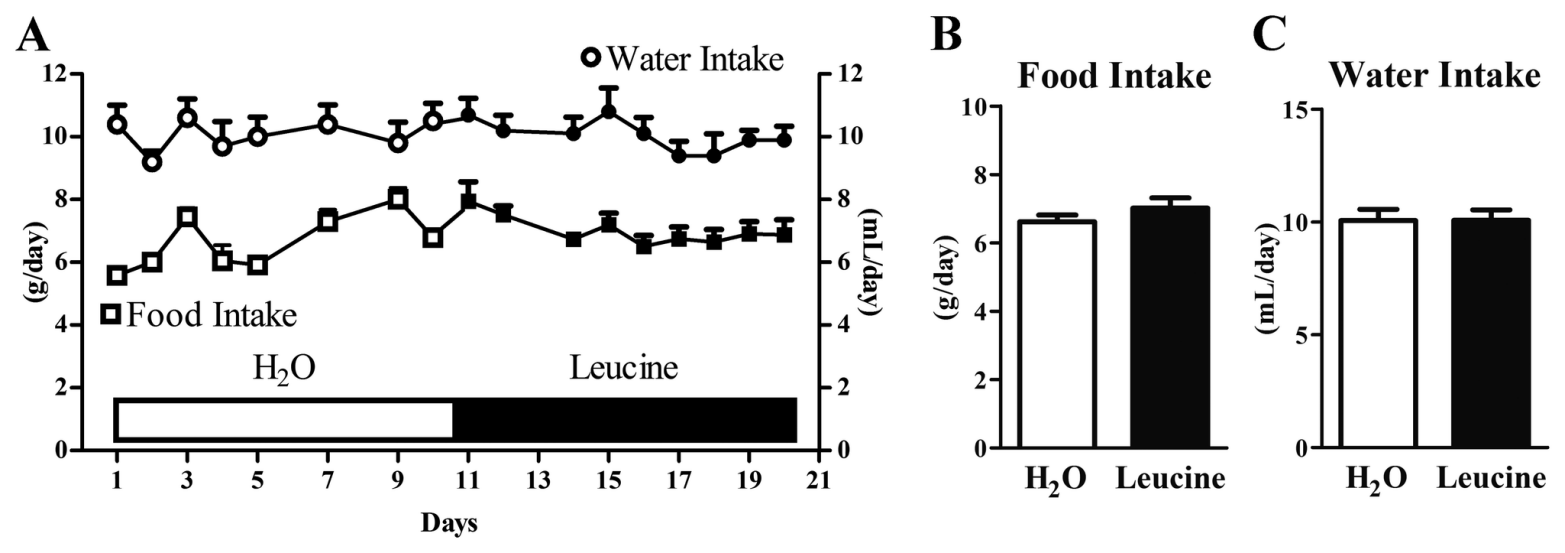
Chronic leucine supplementation did not change the food intake and water ingestion of ob/ob mice. The food intake and water ingestion of ob/ob mice (*n* = 7) were assessed for 20 consecutive days. During the first 10 days, the mice received water to drink (H_2_O, white bar), followed by 10 days of leucine supplementation in the drinking water (Leucine, black bar).

 We assessed the food and water intake of *ob/ob* mice for 20 days. In the first 10 days, the mice received water to drink. In the remaining days, they were supplemented with leucine in the drinking water. Each mouse acted as its own control by comparing their food and water intake before and during the supplementation period. The food intake of *ob/ob* mice remained unaltered during the supplementation period compared to the food intake assessed when the mice received only water to drink (Figure 4A-B). Water intake also was not affected by leucine supplementation (Figure 4C). Similarly, weight gain was not influenced by leucine supplementation (Control: 0.28 ± 0.02 g/day; Leucine: 0.27 ± 0.03 g/day; *P* = 0.7273). Based on the amount of leucine obtained through the diet and water, total leucine intake during the supplementation period was 2.7-fold (0.32 ± 0.01 g/day) higher than the basal period (0.12 ± 0.01 g/day). 

### Experiment 5

#### Chronic leucine supplementation in the drinking water does not change the food intake of wild-type mice receiving a low- or a high-fat diet

**Figure 5:**
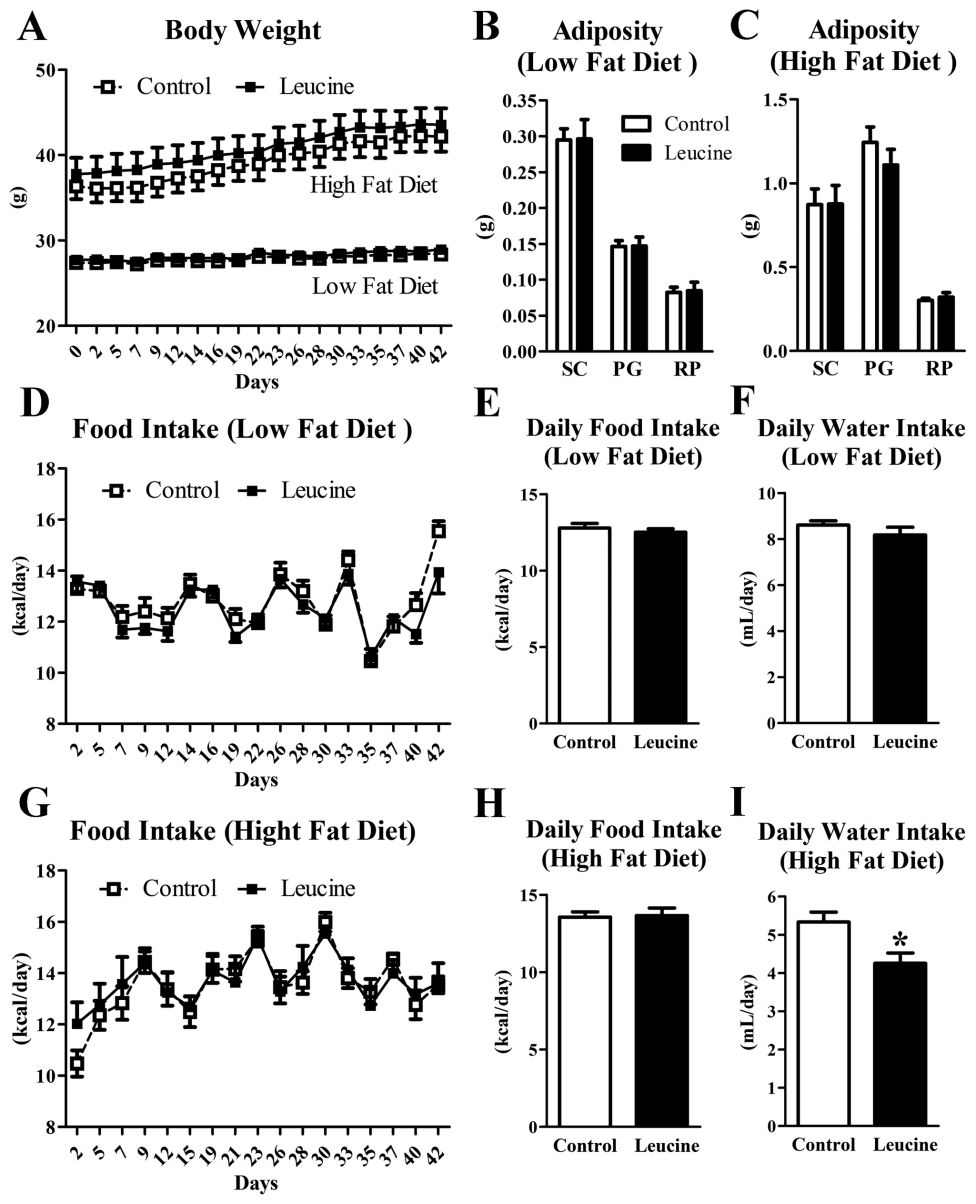
Body weight, adiposity and food intake were not affected by chronic leucine supplementation. Body weight (A), mass of subcutaneous (SC), perigonadal (PG) and retroperitoneal (RP) fat pads (B-C), food intake (D-E, G-H) and water consumption (F, I) in mice receiving low- and high-fat diets of control and leucine groups (*n* = 9-10 per group). *, significantly different (*P* < 0.05) from control group.

 Now we decided to study the effects of leucine supplementation in the drinking water of wild-type mice receiving either a low- or a high-fat diet (HFD). Initially, a group of mice received a HFD for 2 months while another group was kept in their regular low-fat diet. Then, half of the mice in each group received leucine supplementation in the drinking water for 6 weeks, whereas the other half had water to drink. The consumption of the HFD caused a marked increase in the body weight (Figure 5A) and adiposity (Figure 5B-C) compared to mice consuming the regular low-fat diet. Oral leucine supplementation did not change the body weight of mice receiving either a low- or a high-fat diet (Figure 5A). In accordance, no changes in the mass of subcutaneous, perigonadal and retroperitoneal fat pads were observed between control and leucine groups (Figure 5B-C). We assessed the food intake all over the experiment and we failed to find significant differences in the calorie intake comparing control and leucine groups in both diets (Figure 5 D-E,G-H). Water intake remained unchanged in leucine-supplemented mice consuming a low-fat diet (Figure 5F). However, leucine supplementation in the drinking water reduced the amount of liquid ingested by mice consuming the HFD (Figure 5I). We assessed serum corticosterone levels to determine whether chronic leucine supplementation in the drinking water caused a stress response (Figure S1). No changes were observed between control and leucine groups, either in mice consuming low- or high-fat diets (Figure S1). In addition, corticosterone levels were 4 to 7 fold lower in mice of the control and leucine groups compared to animals that were subjected to conditions that increase corticosterone secretion such as psychosocial stress or prolonged fasting (Figure S1). For the mice consuming the low-fat diet, leucine supplementation in the drinking water increased total leucine intake 3-fold (0.23 ± 0.01 g/day) compared to the control group (0.08 ± 0.00 g/day). In the mice consuming the HFD, leucine intake increased approximately 2.3-fold in the supplemented group (0.10 ± 0.00 g/day) compared to the control HFD group (0.05 ± 0.00 g/day).

Lack of evidence that leucine supplementation induces an anorectic pattern of gene expression in the hypothalamus, despite significant effects on the expression of enzymes that metabolize BCAA

**Figure 6:**
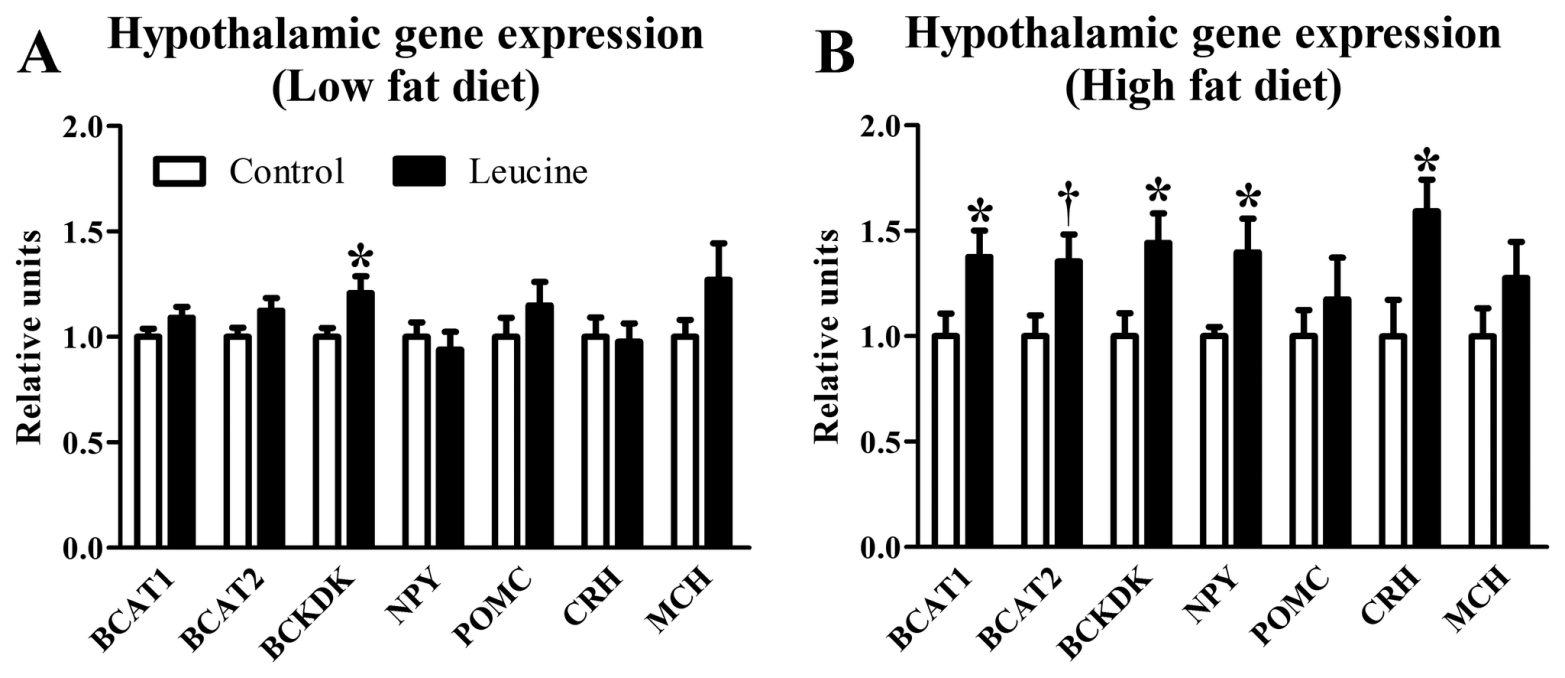
Hypothalamic mRNA expression of genes involved in the BCAA metabolism and energy balance regulation. Gene expression was assessed in mice receiving a low-fat diet (A, *n* = 10 per group) or a high-fat diet (B, *n* = 9-10 per group). *, significantly different (*P* < 0.05) from control group. †, *P* = 0.0532 versus control group.

 The mice from the previous experiment were euthanized, and their hypothalami collected for gene expression analysis. Initially, we assessed whether leucine supplementation in the drinking water was able to affect the expression of genes that encode the enzymes that metabolize the BCAA. The BCAT1 gene, which encodes the cytosolic form of the enzyme branched-chain amino acid transaminase and is highly expressed in the brain [43], and the BCAT2 gene, which encodes the mitochondrial form of the enzyme branched-chain amino acid transaminase, showed no changes in mice consuming a low-fat diet, but a higher expression in leucine-supplemented animals consuming the HFD (Figure 6A-B). The branched-chain α-ketoacid dehydrogenase complex (BCKDK) showed an increased expression in the leucine-supplemented groups, regardless of diet (Figure 6A-B).

Previous studies have demonstrated that central leucine administration causes a rapamycin-dependent anorectic behavior, possibly through its influence on the hypothalamic circuitry that regulates energy balance [22,30,38,39,44]. However, much less is known about how chronic oral leucine supplementation can affect the pattern of gene expression in the hypothalamus. Therefore, we assessed the expression of several genes involved in the regulation of food intake. Chronic leucine supplementation in mice consuming the HFD caused an increased mRNA expression of the neuropeptide Y (NPY) and corticotropin-releasing hormone (CRH, Figure 6B). No changes in mRNA expression of pro-opiomelanocortin (POMC) and melanin-concentrating hormone (MCH) were observed with leucine treatment (Figure 6A-B).

## Discussion

Leucine supplementation has received special attention because of its possible effects on the regulation of energy balance and food intake [13,17,18,30]. Herein, we studied chronic oral supplementation of leucine in genetically obese mice and in wild-type mice receiving either low- or high-fat diets. Overall, we failed to observe any significant reduction on food intake or in the induction of an anorectic pattern of gene expression in the hypothalamus. However, in an acute paradigm of leucine supplementation, leucine seems to increase food intake. In addition, we collected several pieces of evidence showing that the brain is in fact able to sense the increased ingestion of leucine, by increasing the hypothalamic phosphorylation of p70S6K and the activation of neurons in the AP, and by changing the expression of several genes in the hypothalamus.

Our group and others have studied leucine supplementation in rodents in distinct metabolic situations, such as food restriction [19,31,32], aging [13,20,34,45,46], protein-restricted diets [33,47], high-caloric diets [21-27,35,37,48,49] and genetically obese models [22,50]. In the vast majority of these studies, oral leucine supplementation, provided either in the diet or in the drinking water, caused no changes in food intake [13,14,19-21,24-27,31-37,45-50], although there are exceptions [22,23,38,51]. In contrast, studies that aimed to assess possible effects of leucine on the CNS and therefore infused leucine directly into the brain have consistently shown reductions in food intake [22,30,38,39,44]. Why, then, does oral leucine supplementation usually result in different effects on regulation of food intake compared to centrally injected leucine? By infusing leucine directly into the brain, it bypasses the blood-brain barrier and other “natural filters” (i.e., glia cells, tanycytes, etc.) that protect the brain from toxic agents or strong variations in substances in the blood. Therefore, it is not surprising that central leucine administration produces a much stronger effect on brain function compared to paradigms that provide leucine through the diet or drinking water. However, the fact that oral leucine supplementation in most of the studies cannot recapitulate the reduction in food intake observed after centrally infused leucine suggests that these presumable effects of leucine must be revised or examined with caution. It is important to consider that if leucine becomes a therapeutic supplement to treat obesity or other diseases in human beings, just as it has been tested [28], leucine would be provided in an oral form. Therefore, it would be more useful for future studies that assess the effects of leucine on the regulation of food intake focus primarily on oral forms of supplementation.

There is evidence that leucine-rich diets may induce taste aversion in rodents, at least temporarily [22,38]. Besides, in excess leucine can be toxic [52]. To avoid possible interference of leucine in the food palatability, we decided to supplement leucine in the drinking water, which resulted in no changes in food intake either in genetically obese mice or in wild-type mice. The limitation is the relatively low solubility of leucine in water and the fact that leucine supplementation will be linked with water ingestion. Despite that, we were able to increase daily leucine intake by up to 3-fold compared to control animals. It is worth mentioning that the increase in leucine intake attained by providing it in the drinking water was higher or equivalent to the supplementation dose used in several previously published studies that showed significant effects of leucine [19,21,24-26,31,32,35,36,47,49,50]. 

The studies that found changes in food intake mostly provided leucine supplementation through the diet [22,23,38]. In one study, leucine-induced taste aversion was observed only on the first day of supplementation [22]. However, Koch et al. [38] found that dietary leucine supplementation reduced the food intake by inducing taste aversion, whereas leucine supplementation in the drinking water caused no effects on food intake. In addition, only i.c.v. administration of leucine reduced food intake, whereas gavage, subcutaneous or intraperitoneal infusions caused no changes in feeding behavior [38]. Because dietary leucine supplementation may have this confounder, it is also important that future studies perform the required controls to rule out possible effects of leucine on food intake through influencing taste aversion or food palatability.

Previous studies found that the central infusion of leucine causes p70S6K phosphorylation in brain areas related to the regulation of food intake, including the ARH, PVH, and NTS, but not in the VMH, zona incerta, hippocampus and cerebral cortex [22,30,44]. In addition, the infusion of leucine in the mediobasal hypothalamus not only induces Fos expression in the ARH but also in areas outside the injection site, such as the PVH and NTS [39]. We failed to detect significant changes in Fos expression in the PVH, ARH or NTS. Another study [53] identified amino acids that are able to induce Fos expression in the hypothalamus. The authors found that the nonessential amino acid asparagine induces Fos expression in orexin neurons, which are involved in the regulation of food intake, whereas leucine does not [53]. We observed a significant increase in Fos expression only in the AP. The AP is considered an important area for chemoreception, both in detecting toxins and controlling nausea and vomiting. Lesions of AP prevent taste-aversion conditioning after administration of lithium chloride [54,55] or other substances [56]. Therefore, the AP may be involved in the brain´s ability to sense leucine intake.

It has long been known that variations in leucine availability induce changes in the expression and/or activity of enzymes that metabolize BCAA [57-60]. Much less is known about the influence of leucine supplementation on the hypothalamic expression of these enzymes. Some authors suggest that the high expression of the cytosolic form of BCAT in the brain plays a role in the synthesis of amino acid neurotransmitters (e.g., glutamate and GABA) by transferring the nitrogen group from BCAA to neurotransmitter precursors [61]. Furthermore, cytosolic BCAT may also be involved in the ability of the brain to sense the availability of amino acids [61]. Regarding our results, we observed an increased expression of BCAT1, BCAT2 and BCKDK in the groups supplemented with leucine, particularly in mice consuming the HFD. The increased expression of the enzymes that metabolize BCAA in leucine-supplemented animals is in accordance with the fact that the body must prevent excessive increases in circulating leucine levels, lest it cause toxic effects. BCAT2-knockout mice display very high levels of BCAA and a lean phenotype which is associated with elevated energy expenditure [62]. Interestingly, BCAT2-knockout mice have increased food intake, most likely as a compensatory mechanism for the higher energy expenditure [62]. In accordance, other studies also observed increased energy expenditure in animals supplemented with leucine [21,25], sometimes associated with increased food intake [21]. So, it is possible that the increased food intake observed after acute administration of leucine in our study was caused by compensatory mechanisms seeking to reestablish the energy balance. Leucine supplementation did not affect the expression of genes involved in the regulation of the energy balance in mice consuming a low-fat diet. However, we observed an increased expression of the orexigenic NPY in diet-induced obese mice, at the same time they exhibited an increased expression of the anorexigenic CRH. The reasons for this particular pattern of hypothalamic gene expression are unknown, but it may also reflect an attempt of the organism to defend the energy homeostasis by balancing orexigenic and anorexigenic neuronal pathways. In the long-term, the resulting consequence may be the lack of changes in food intake as observed in our study. Lastly, it is important to consider that leucine supplementation may produce significant effects on body metabolism independently of changes in the food intake. These effects can be observed in studies that found reductions in adiposity in animals supplemented with leucine, even in the absence of changes in food intake [19-21,24,25,31,37].

In summary, three sets of experiments using different methods (activation of intracellular pathways, induction of Fos protein and analysis of gene expression) demonstrated that the brain possesses mechanisms allowing it to detect changes in the availability of leucine from oral intake. Thus, leucine, as well as glucose and lipids [3-7], can be sensed by the brain. We also suggest that the AP is a putative area involved in the brain’s leucine chemoreception. Despite the fact the brain is able to sense leucine, chronic oral leucine supplementation (in the drinking water) does not produce changes in the food intake, nor does it cause an anorectic pattern of gene expression in the hypothalamus. Although future studies should directly assess the consequences of leucine supplementation in humans, our results question the possible efficacy of leucine supplementation as an appetite suppressant to treat obesity.

## Materials and Methods

### Animals

The mice used in the experiments were maintained under standard conditions of light (12 h light/dark cycle; lights on 8:00 am), temperature (22 ± 2 °C) and relative humidity (55 ± 15%). All animal procedures were approved by the Ethics Committee on the Use of Animals of the Institute of Biomedical Sciences, University of São Paulo, and were performed according to the ethical guidelines adopted by the Brazilian College of Animal Experimentation.

### Experiment 1

 Single-housed 10-week-old C57BL/6 male mice were daily habituated to receive gavage for one week. Then, mice were fasted overnight and at 9:00 am received an oral gavage administration of 0.5 mL of water (control group, *n* = 9) or 0.15 м l-leucine (Synth, Brazil) solution (leucine group, *n* = 9). The chosen dose of l-leucine (0.15 м or 1.967% wt/vol) is near to its limit of solubility in water. Previous studies used this same dose in the drinking water [26] or slightly lower amounts (1.5-1.7% wt/vol) [21,24,25,35,38]. The mice were euthanized by decapitation 90 min after the oral gavage, and the hypothalami were quickly collected for western blot analysis. To dissect the hypothalamus, we defined its limits as follows: rostro-caudal, 1 mm anterior to the optic chiasm and immediately posterior to the mammillary bodies; lateral, defined by the optic tract; and superior, the dorsal limit of the third ventricle.

### Experiment 2

 Single-housed 10-week-old C57BL/6 mice were habituated daily to handling and to receiving oral gavage for one week to minimize unspecific Fos expression. After that, mice were fasted for 4 h, followed by an oral gavage administration of 0.5 mL of water (control group, *n* = 10) or 0.15 м L-leucine solution (leucine group, *n* = 9) at 1:00 pm. After 3 h, mice were deeply anesthetized and perfused transcardially with saline followed by a 4% formaldehyde solution (150 mL each mouse). Brains were collected and post-fixed in the same fixative for 2 h and cryoprotected overnight at 4 °C in 0.1 м phosphate-buffered saline (PBS), pH 7.4, containing 20% sucrose. Brains were cut (30-µm sections) in the frontal plane using a freezing microtome. Four series were collected in antifreeze solution and stored at -20 °C. The time chosen to perfuse the mice after the oral gavage of leucine to detect Fos immunoreactivity (Fos-ir) was based on previous studies [53].

### Experiment 3

 Single-housed 10-week-old C57BL/6 male mice (*n* = 20) were habituated daily to handling and to receiving oral gavage (0.5 mL of water) for one week. Then, we assessed for 4 consecutive days their 24 h food intake and body weight. In the first 2 days, mice received an oral gavage administration of 0.5 mL of water. In the next 2 days, they received an oral gavage administration of 0.15 м L-leucine solution. The gavage took place 3 hours before lights off. We assessed whether leucine supplementation acutely affect the food intake, weight gain and body weight of mice in comparison with the days the mice received water.

### Experiment 4

 Ten-week-old C57BL/6 leptin-deficient (*ob/ob*) mice (*n* = 7; initial body weight: 39.6 ± 0.5 g) were acclimated to single housing for 1 week. After that, their food intake and water ingestion were assessed daily for 20 consecutive days. In the first 10 days of the experiment, the mice received water to drink. In the remaining days, the contents of the drinking bottle were replaced with a 0.15 м L-leucine solution. During the entire experiment, the mice had ad libitum access to a regular rodent chow diet (Quimtia, Nuvilab CR-1, Brazil). The objective of this experiment was to assess whether leucine supplementation changes the food intake in comparison to the data collected when the mice received only water to drink. The purpose in using the same animal as reference (control) was to minimize interindividual variability in assessing the effects of leucine on food intake. Therefore, each mouse acted as its own control.

### Experiment 5

Eight-week-old C57BL/6 male mice (*n* = 39) were distributed into two groups according to diet: low-fat regular rodent chow diet (Quimtia, Nuvilab CR-1, Brazil; 2.99 kcal/g; 9.4% calories from fat) or a high-fat diet (PragSoluções, Brazil; 5.31 kcal/g, 58% calories from fat). After 2 months, each group was redistributed into two more groups: control and leucine groups. The mice were kept on their original diets, but the leucine groups received a 0.15 м leucine solution in the drinking water, whereas the control groups had water to drink. During the experiment, the mice had ad libitum access to their experimental diets and drinking bottle and we assessed their food intake, water ingestion and body weight 3 times per week. After 6 weeks of supplementation, the mice were fasted for 4 h and euthanized by decapitation. The hypothalami were dissected as previously described and frozen at -80 °C for gene expression analysis. Serum corticosterone levels were assessed by ELISA (Cayman Chemical).

### Western Blot

Immediately after collection, the hypothalami were homogenized in RIPA buffer (Sigma) containing a cocktail of protease and phosphatase inhibitors (1:100, Sigma), then resolved in 10% SDS-PAGE gel and finally transferred to a nitrocellulose membrane. After blocking the membrane with 5% BSA, the membrane was incubated overnight at 4°C using commercially available antibodies (phospho-p70S6K, Cell Signaling, #9205, 1:1,000; and α-actinin, Santa Cruz, H-300, 1:1,000). Next, we incubated the membrane for 45 min in IRDye 800CW secondary antibody (1:10,000, Li-COR). Proteins were visualized and analyzed using the Li-COR Odyssey system (Li-COR) and phospho-p70S6K expression was normalized to α-actinin expression.

### Immunohistochemistry

The immunohistochemistry protocol followed has been previously described [63]. Briefly, brain sections were rinsed in 0.02 м potassium PBS, pH 7.4 (KPBS), followed by a pretreatment with 0.3% hydrogen peroxide for 30 min. After rinses in KPBS, sections were blocked in 3% normal donkey serum for 1 h, followed by incubation in anti-Fos polyclonal primary antibody raised in rabbit (1:20,000, Ab5, Millipore) for 48 h. Subsequently, sections were incubated for 1 h in biotin-conjugated IgG donkey anti-rabbit (1:1,000, Jackson Laboratories) and for 1 h in avidin-biotin complex (1:500, Vector Labs). The sections were then submitted to a 3-5 min immunoperoxidase reaction with 0.03% hydrogen peroxide dissolved in 0.1 м acetate buffer, pH 6.0, using diaminobenzidine tetrahydrochloride (DAB; Sigma) and 0.5% nickel sulfate as chromogens. Sections were mounted onto gelatin-coated slides, dried overnight, dehydrated in ethanol, cleared in xylene and coverslipped with DPX Mountant for histology (Sigma-Aldrich). Fos-ir was analyzed in the PVH, ARH, VMH, LHA, DMH, NTS and AP. Nuclear boundaries were determined using a mouse brain atlas as reference [64]. We counted the number of cells expressing a dark-brown/black nuclear staining in one side of a representative rostral-to-caudal level of each area. A researcher with experience in counting Fos immunoreactivity and blinded to the experimental groups was designed to do all the counting. The ImageJ Cell Counter tool was used to mark each counted cell avoiding double counting. Photomicrographs were acquired with a Zeiss Axiocam HRc camera adapted to a Zeiss Axioimager A1 microscope (Zeiss, Munich, Germany). Images were digitalized using the Axiovision software (Zeiss). Photoshop CS5 (Adobe) image-editing software was used to combine photomicrographs into plates. Only sharpness, contrast and brightness were adjusted.

### Relative Gene Expression (qPCR)

Total RNA were extracted with TRIzol® reagent (Invitrogen) according to the manufacturer’s instructions. Assessment of RNA quantity and quality was performed with an Epoch Microplate Spectrophotometer (Biotek®). Total RNA was incubated in DNase I RNase-free (Roche Applied Science). Reverse transcription was performed with 2 µg of total RNA with SuperScript® II Reverse Transcriptase (Invitrogen) and random primers p(dN)6 (Roche Applied Science). Real-time polymerase chain reaction (qPCR) was performed using the 7500 Fast Real-Time PCR System (Applied Biosystems®) on duplicates of each cDNA and optimized using Power SYBR Green PCR Master Mix or TaqMan® Gene Expression Master Mix (both from Applied Biosystems®). Speciﬁc primers were designed for each target gene according to sequences taken from GenBank or the literature (Table S1). Melt curve analysis was conducted to validate the speciﬁcity of the primers. Relative quantification of mRNA was calculated by 2^-ΔΔCt^ [65]. Data were normalized to β-actin expression and reported as fold changes compared to values obtained from the control group (set at 1.0).

### Statistical analysis

For statistical analysis of the data from Experiments 1, 2 and 5, we used the unpaired two-tailed Student’s *t*-test. In Experiment 3, data were compared using the repeated measures ANOVA followed by Newman-Keuls posttest. In Experiment 4, data were compared using the paired two-tailed Student’s *t*-test. A P value of <0.05 was considered significant in all analyses. The results are expressed as the mean ± SEM. Statistical analysis was performed using GraphPad Prism software.

## Supporting Information

Figure S1 **Serum corticosterone levels.** Corticosterone levels of control and leucine groups (*n* = 8-10 per group) were compared to values obtained from mice that were subjected to psychosocial stress (3 days in individual cages followed by regrouping for 30 min; *n* = 4) or prolonged fasting (*n* = 8). *, significantly different (*P* < 0.05) from control and leucine groups.
(TIF)

Table S1 **Primer sequences.**
(DOCX)
