# Supplementary figures and images for: Oral Leucine Supplementation Is Sensed by the Brain but neither Reduces Food Intake nor Induces an Anorectic Pattern of Gene Expression in the Hypothalamus
Source: PLoS One. 2013 Dec 13;8(12):e84094. doi: 10.1371/journal.pone.0084094 (PMC3862776; doi:10.1371/journal.pone.0084094)

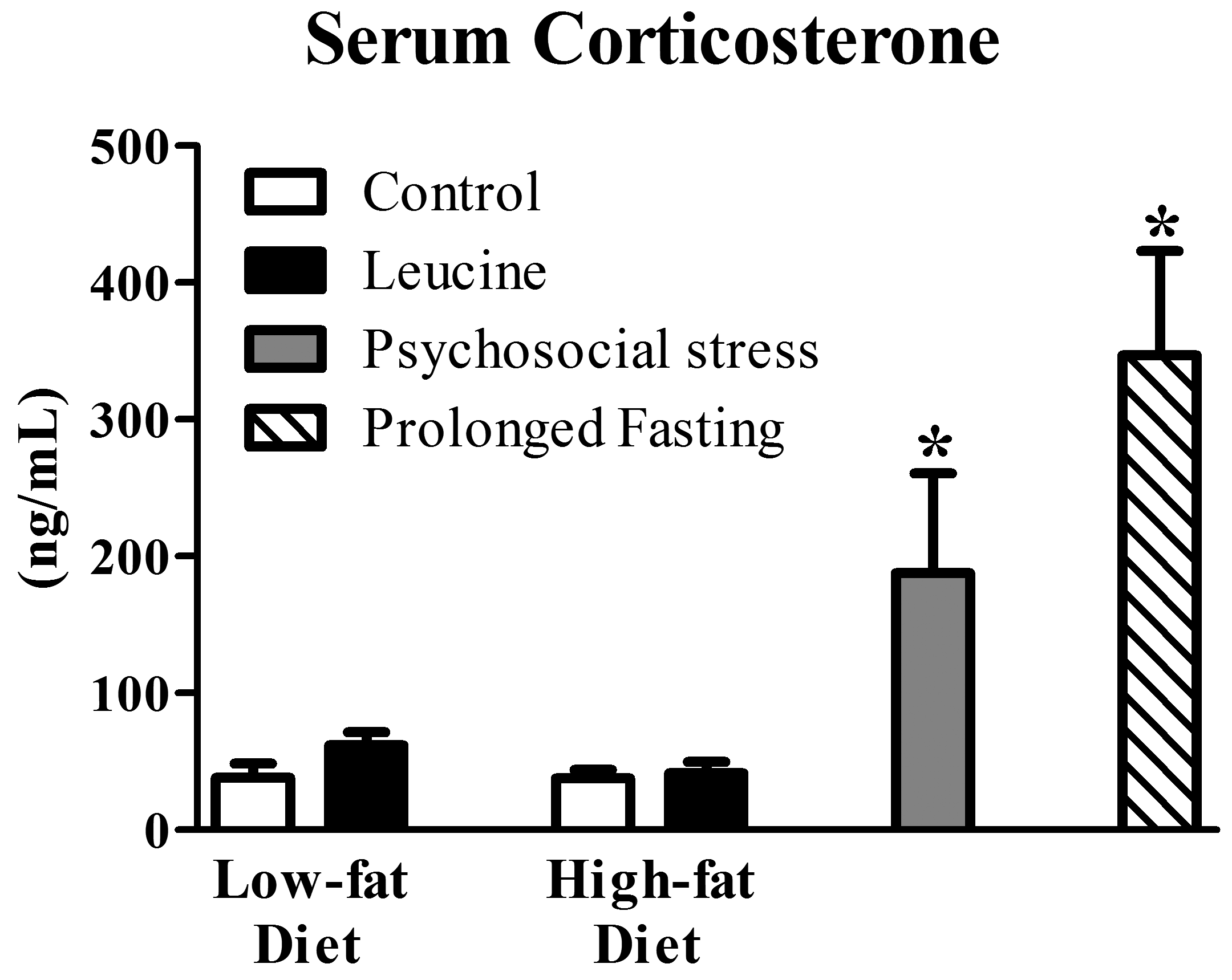

Supplement: Figure S1 — Serum corticosterone levels. Corticosterone levels of control and leucine groups (n = 8-10 per group) were compared to values obtained from mice that were subjected to psychosocial stress (3 days in individual cages followed by regrouping for 30 min; n = 4) or prolonged fasting (n = 8). *, significantly different (P < 0.05) from control and leucine groups. (TIF) [file pone.0084094.s001.tif]
