# Supplementary material for: Oral Leucine Supplementation Is Sensed by the Brain but neither Reduces Food Intake nor Induces an Anorectic Pattern of Gene Expression in the Hypothalamus
Source: PLoS One. 2013 Dec 13;8(12):e84094. doi: 10.1371/journal.pone.0084094 (PMC3862776; doi:10.1371/journal.pone.0084094)
Supplement: Table S1 — Primer sequences. (DOCX) [file pone.0084094.s002.docx]

**Table S1** Primer sequences.

| **Gene of interest** | **Accession no.** | **Forward primer (5’-3’)** | **Reverse primer (5’-3’)** | **Product size (bp)** |
| --- | --- | --- | --- | --- |
| β-Actin^*^ | Mm00607939_s1 | - | - | - |
| BCAT1 | NM_001024468.3 | TGTGCCACGTATTCCCACG | CTCTTTCTCCAGCAAACGGC | 120 |
| BCAT2 | NM_001243052.1 | TCCATGACACCTGTCTCCCT | CAGCCACAGTGGGTCCATAG | 106 |
| BCKDK | NM_009739.3 | TGATGCTCTATTCCGGTCGC | TTGATGCGGTGAGCAATCCT | 100 |
| NPY^*^ | Mm03048253_m1 | - | - | - |
| POMC | NM_001278581.1 | GAGGCCACTGAACATCTTTGTC | GCAGAGGCAAAACAAGATTGG | 253 |
| MCH | NM_029971.2 | CCAGCTGAGAATGGAGTTCAGA | GTCGGTAGACTCTTCCCAGCAT | 139 |
| CRH | NM_205769.2 | TGGATCTCACCTTCCACCTTCTG | CCGATAATCTCCATCAGTTTCCTG | 103 |

^*^, TaqMan® Gene Expression Assay (Applied Biosystems).
